# Endobronchial ultrasound-guided transbronchial needle aspiration as a diagnostic modality for schwannoma: A case report

**DOI:** 10.1097/MD.0000000000029669

**Published:** 2022-07-22

**Authors:** Wei Liu, Yun Li, Lingjia Chen, Zhiguang Liu, Weidong Zhang

**Affiliations:** a Department of Pulmonary and Critical Care Medicine, Hunan Provincial People’s Hospital, The First Affiliated Hospital of Hunan Normal University, Changsha, Hunan, People’s Republic of China.

**Keywords:** case report, diagnosis, endobronchial ultrasound-guided transbronchial needle aspiration, schwannoma

## Abstract

Schwannomas are the most common nerve sheath tumors in the paravertebral mediastinum. Although radiological imaging is helpful in diagnosing schwannomas, a definitive diagnosis is dependent on pathological features of a surgical specimen. For patients who require preoperative diagnosis, an incisional biopsy using minimally invasive surgery is preferred. Endobronchial ultrasound-guided transbronchial needle aspiration (EBUS-TBNA) is less commonly used for suspected schwannomas.

**Patient concerns:**

A 48-year-old woman presented to the clinic with complaints of productive cough for >1 month, expectorating yellow and mucoid sputum approximately 4 to 5 times per day. Chest computed tomography revealed a well-circumscribed, homogeneous, soft tissue mass lesion in right upper posterior mediastinum, measuring 55 mm × 44 mm. Vocal fremitus in the right upper lung was diminished, the percussion note was slightly dull, and breath sounds were slightly reduced on auscultation. The patient was a nondrinker and nonsmoker, with no other relevant medical history. There was no significant relevant family medical history.

**Diagnosis:**

Complete blood count and blood biochemistry were within normal limits, except for an elevated erythrocyte sedimentation rate (32 mm/h). EBUS-TBNA was performed and histopathological findings were consistent with schwannoma.

**Interventions:**

The patient underwent schwannoma excision by thoracoscopy. Pathological findings from the surgical specimen were consistent with the EBUS-TBNA results. Based on EBUS-TBNA and postsurgical pathology, the patient was diagnosed with a right upper mediastinal schwannoma (Antoni B).

**Outcomes:**

The patient experienced an uneventful postoperative recovery with no adjuvant therapy and was discharged on April 18, 2017. The patient has been followed up for 4 years and has not experienced any symptoms.

**Conclusions:**

Cell blocks obtained from EBUS-TBNA afford the possibility of cytological examination and immunocytochemical staining, which can confirm diagnosis of schwannoma.

## 1. Introduction

Neurogenic tumors are the most common neoplastic lesions of the paravertebral mediastinum, accounting for approximately 53.9%^[1]^ to 96.1%^[2]^ of paravertebral mediastinal lesions. Schwannomas and neurofibromas are the most common nerve sheath tumors. Most schwannomas, however, are benign and usually grow slowly; as such, for a malignant peripheral nerve sheath tumor to arise from a schwannoma is extremely rare.^[3]^ Surgical excision is the preferred treatment for schwannomas^[2,4]^ and the prognosis is favorable, with a typical 5-year survival rate of 97% after surgery.^[5]^

The use of computed tomography (CT), magnetic resonance imaging (MRI), and/or positron emission tomography-CT (PET-CT) enables a precise preoperative diagnosis and the design of an appropriate surgical treatment strategy. Preoperative diagnosis facilitates disease management and prompts further clinical measures. For patients who require surgical treatment, a definitive diagnosis can guide the choice of surgical approach.^[2,4,6]^ Endobronchial ultrasound-guided transbronchial needle aspiration (EBUS-TBNA) has conventionally been used to diagnose mediastinal and hilar lesions and to stage malignancies,^[7]^ but is less commonly used for neurogenic tumors. In particular, the use of EBUS-TBNA to diagnose schwannomas has not been frequently reported.^[8–12]^ Herein, we report a case involving a patient with schwannomas diagnosed using EBUS-TBNA and confirmed by pathological analysis of surgical specimens.

## 2. Case Report

**Figure 1, 2.**
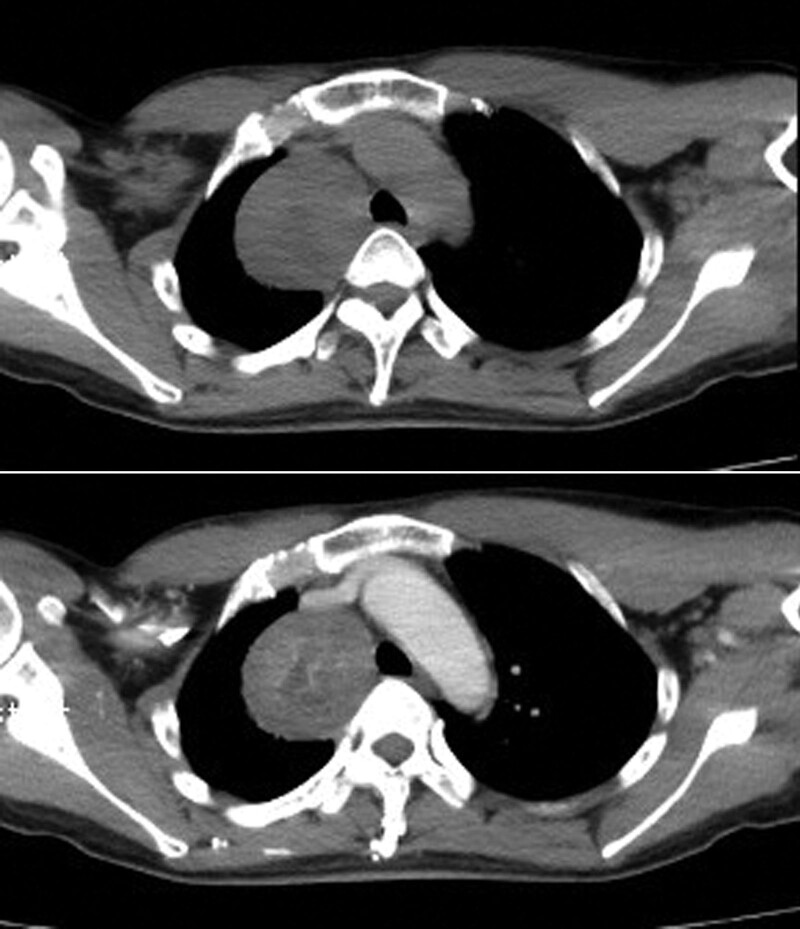
Chest computed tomography revealing a well-circumscribed, homogeneous, soft tissue mass lesion in the right upper posterior mediastinum, contrast CT showed mildly heterogeneous enhancement.

A 48-year-old woman with a >1-month history of cough and expectorating sputum presented to the hospital. The patient complained of cough for >1 month, associated with expectoration of yellow mucoid sputum approximately 4 to 5 times per day, but without fever, chest pain, shortness of breath, or malaise. Chest CT performed on March 22, 2017, revealed a well-circumscribed, homogeneous, soft tissue mass lesion in right upper posterior mediastinum, measuring 55 mm × 44 mm, and mildly heterogeneous enhancement on contrast (Figs. 1 and 2). A neurogenic tumor was suspected. Since the onset of symptoms, the patient experienced weight loss of approximately 2.5 kg. Physical examination yielded the following findings: blood pressure 109/81 mm Hg, heart rate 99 beats/min, respiratory rate 20 breaths/min, oxygen saturation 99%, oxygen concentration 0.21, and temperature 36.5°C. No palpable lymph nodes were reported. The trachea was central. Vocal fremitus of the right upper lung was diminished, and the percussion note was slightly dull with breath sounds slightly reduced on auscultation, and there were no dry or wet crackles or sounds of pleural friction rub heard. Physical examination of other systems yielded unremarkable findings. The patient was a nondrinker and nonsmoker, with no other relevant medical history. There was no significant relevant family medical history.

**Figure 3, 4.**
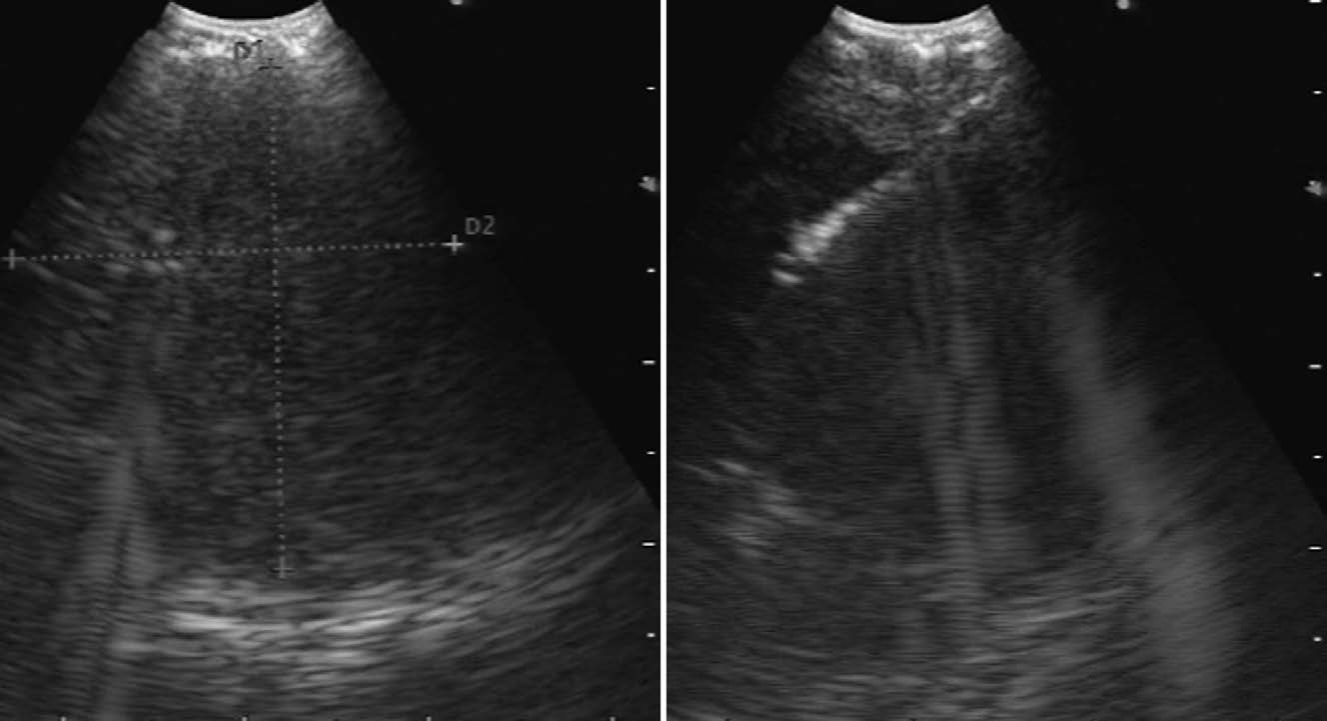
Endobronchial ultrasound revealing a well-defined, round mass with a homogeneous echogenic nature. Transbronchial needle aspiration was performed.

**Figure 5.**
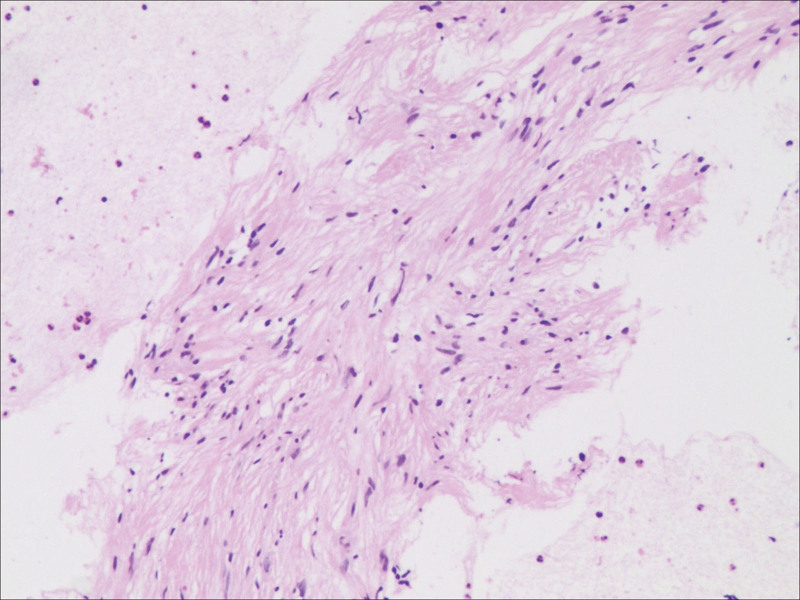
Endobronchial ultrasound-guided transbronchial needle aspiration specimen. Scattered focal spindle tumor cells, with no atypia and no mitotic signs, are evident on the background of loose edematous and mucinous stroma with fibrillar collagen (hematoxylin and eosin stain, original magnification ×20).

**Figure 6.**
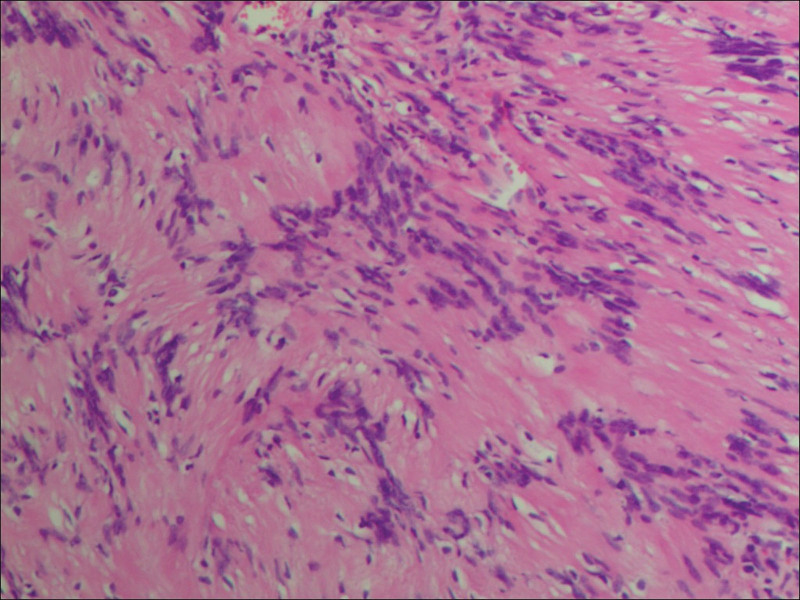
Surgical specimen. Tumor cells are spindle-shaped and fenestrated with eosinophilic cytoplasm (hematoxylin and eosin stain, original magnification ×20).

Complete blood count and blood biochemistry results were within normal limits, except for an elevated erythrocyte sedimentation rate (32 mm/h). A coagulation function test was in the normal range and tumor markers were negative. Sputum smear and culture, as well as sputum acid fast staining smear, were negative. Electrocardiography findings suggested sinus arrhythmia. Bronchoscopy revealed no abnormalities. EBUS revealed a well-circumscribed, round, homogeneous echogenic mass at the upper right paratracheal area, and TBNA was performed (Figs. 3 and 4). Histopathological findings revealed scattered focal spindle tumor cells, with no atypia and no mitotic signs on a background of loose edematous and mucinous stroma with fibrillar collagen. Immunohistochemical staining yielded the following findings: vimentin positive (+), S-100 (+), cluster of differentiation 56 (+), smooth muscle actin (–), cytokeratin (pan) (–); Ki-67 (+ [5%]), and periodic acid schiff (–). These findings were consistent with schwannoma (Fig. 5). The patient underwent mass excision via thoracoscopy. Pathological findings from the surgical specimen were consistent with EBUS-TBNA results (Fig. 6). Based on EBUS-TBNA and postsurgical pathology, the patient was diagnosed with a right upper mediastinal schwannoma (Antoni B). The patient experienced an uneventful postoperative recovery with no adjuvant therapy and was discharged on April 18, 2017. The patient has been followed up for 4 years and has not experienced any symptoms.

## 3. Discussion

Mediastinal lesions are rarely encountered. The prevalence of mediastinal lesions ranges from 0.73% to 0.9%.^[13–15]^ The prevascular mediastinum is the most common compartment where these lesions occur (69.8%), followed by the visceral (13.5%) and paravertebral (5.4%) mediastinum, although some lesions have been found within 2 or 3 compartments.^[1]^ Neurogenic tumors are the most common neoplastic lesions of the paravertebral mediastinum, accounting for approximately 53.9%^[1]^ to 96.1%^[2]^ of lesions. Neurogenic tumors arise from tissues originating in the embryonic neural crest and can be further classified based on whether these grow from the nerve sheath, nerve cells, or paraganglia. Schwannomas and neurofibromas are the most common tumors originating from the nerve sheath. Most schwannomas are benign and grow slowly. A malignant peripheral nerve sheath tumor arising from a schwannoma is extremely rare.^[3]^ Patients are usually asymptomatic when the tumor is small. However, as the tumor increases in size, it may cause compression with adjacent vital organs, nerves, and blood vessels, which may manifest as chest and back pain, Horner syndrome, dysphagia, cough, shortness of breath, hoarseness, wheezing, superior vena cava syndrome, or muscle weakness.^[2,16,17]^ In severe cases, massive hemoptysis may also occur.^[18]^

Imaging studies are important tools in diagnosing and predicting the behavior of schwannomas. Chest X-ray has a limited diagnostic yield for mediastinal lesions. An international, multicenter study investigating the distribution of mediastinal lesions based on a radiological database reported that CT examination was the most commonly used method for the diagnosis of mediastinal lesions.^[1]^ Benign schwannoma on CT is characterized by a round or oval hypodense mass with an envelope and well-defined borders, with a uniform to markedly heterogeneous enhancement on contrast.^[16]^ Margins of the adjacent bone may be sclerosed due to chronic compression. Malignant cases exhibit infiltrative growth with indistinct borders, often with dense heterogeneity within the tumor due to necrosis, hemorrhage, and dystrophic calcification. Occasionally, the tumor degenerates to form a cyst and calcification may occur along the cyst wall.^[17]^ Some tumors grow across the intervertebral foramen, with one side of the tumor located in the mediastinum or thoracic cavity, and the other side in the spinal canal, growing in a dumbbell shape, connected by an enlarged intervertebral foramen, with resorption or destruction of adjacent bone.^[4]^ Schwannomas occurring in the spinal canal are generally well-defined and exhibit characteristic target and nerve access signs, in addition to positive signs of foraminal enlargement.^[19]^ For mediastinal neurogenic tumors that are a challenge to diagnose using CT, MRI can provide useful diagnostic information. In particular, it is a useful diagnostic modality for evaluating the extent of invasion into adjacent structures, characterizing tissue components, such as fat or hemorrhage, and is more effective in differentiating cystic from solid lesions.^[19]^ Schwannomas generally exhibit low or moderate signal on T1-weighted imaging and high signal on T2-weighted imaging, with the T1-weighted imaging and T2-weighted imaging fat suppression sequences being superior to the others and revealing internal tumor pathological changes and tissue features.^[19]^ On enhancement scan, the tumor is heterogeneously enhanced. Diffusion-weighted MRI has been used for the differential diagnosis of neurogenic tumor subtypes. Schwannomas exhibit significantly higher apparent diffusion coefficient values than that of neurofibromas and overlap with apparent diffusion coefficient values of malignant peripheral nerve sheath tumors.^[20]^ Use of ^18^F-Fluorodeoxyglucose PET-CT is recommended by the National Comprehensive Cancer Network guidelines, and is helpful in the evaluation of mediastinal tumors. However, the maximum standard uptake value of schwannomas range from 1.5 to 17.3 with a median of 3.7^[21]^; therefore, definitive diagnosis of schwannoma may be difficult based on SUV profile on PET-CT because maximum standard uptake values appear to vary dramatically.^[22]^

Although radiological imaging is helpful in diagnosing schwannomas, definitive diagnosis remains based on pathological features.^[16]^ Surgical excision is the preferred treatment for schwannomas.^[2,4]^ Preoperative diagnosis facilitates disease management and prompts further clinical measures. For patients who require surgical treatment, a definitive diagnosis will provide guidance for the choice of surgical approach (e.g., thoracotomy, thoracoscopy, cervicotomy, laminectomy). In a study by Lacquet et al,^[2]^ 17 of 51 (33%) patients underwent preoperative biopsy. In general, for cystic lesions and well-encapsulated solid tumors that can be resected primarily, a preoperative tissue biopsy is not required because it will not impact the surgical plan in most cases. However, for mediastinal tumors with suspected malignancy, a biopsy is required to reach a definitive diagnosis to guide the surgical approach.^[2]^ The diagnosis of schwannoma is usually based on findings from examination of the surgical specimen.^[9]^ For patients who require preoperative diagnosis, an incisional biopsy obtained through minimally invasive surgery is preferred.^[2]^ However, some reports have described EBUS-TBNA, endoscopic ultrasound-guided fine-needle aspiration, and transthoracic biopsy for the diagnosis of schwannomas, and diagnosis based on these small specimens is consistent with that of surgical specimens.^[2,8–12,16,23–25]^ When properly prepared, cell blocks obtained from EBUS-TBNA enable cytological examination and immunocytochemical staining, which can confirm the diagnosis of schwannomas. However, when spindle cells are encountered in small specimens (e.g., TBNA), it is difficult to make a definitive diagnosis based on cytological features in some cases because a broad differential diagnosis is needed. Sometimes, small specimens do not reveal immature components of the tumor, which could lead to a false-negative result. In a study by Mlika et al,^[16]^ 2 cases of EBUS-TBNA and 3 cases of transthoracic biopsies failed to obtain an accurate diagnosis. However, EBUS-TBNA enabled us to rule out more frequently encountered mediastinal lesions, such as malignant lymph nodes, lymphoma, thymoma, and cysts.

## 4. Conclusion

Schwannoma is the most common benign neoplastic lesion of the paravertebral mediastinum. Cell blocks obtained from EBUS-TBNA enable cytological examination and immunocytochemical staining, which can confirm the diagnosis of schwannomas. EBUS-TBNA is a helpful method in diagnosing and establishing a treatment strategy for intrathoracic schwannoma.

## Acknowledgments

The authors are grateful to Dr E.K. Makoni, from the Parirenyatwa group of Hospitals in Zimbabwe, for her advice regarding language editing.

## Author contributions

(I) Conception and design: Wei Liu; (II) Administrative support: Weidong Zhang; (III) Provision of study materials or patients: Wei Liu, Zhiguang Liu, Yun Li; (IV) Collection and assembly of data: Wei Liu, Yun Li, Lingjia Chen; (V) Data analysis and interpretation: Wei Liu, Weidong Zhang; (VI) Manuscript writing: All authors; (VII) Final approval of manuscript: All authors
